# Acupuncture and related therapies for anxiety and depression in patients with premature ovarian insufficiency and diminished ovarian reserve: a systematic review and meta-analysis

**DOI:** 10.3389/fpsyt.2024.1495418

**Published:** 2024-12-02

**Authors:** Sidan Huang, Danni Zhang, Xuliang Shi, Yi Zhang, Xuesong Wang, Yanfen She, Ce Liang, Xinyue Li, Christopher Zaslawski

**Affiliations:** ^1^ School of Acupuncture-Moxibustion and Tuina, Hebei University of Chinese Medicine, Shijiazhuang, Hebei, China; ^2^ Hebei International Joint Research Center for Dominant Diseases in Chinese Medicine and Acupuncture, Hebei University of Chinese Medicine, Shijiazhuang, Hebei, China; ^3^ Pharmacological Lab of Traditional Chinese Medicine, Hebei University of Chinese Medicine, Hebei Traditional Chinese Medicine Formula Granule Technology Innovation Center, Shijiazhuang, Hebei, China; ^4^ School of Life Sciences, Faculty of Science, University of Technology Sydney, Sydney, NSW, Australia

**Keywords:** acupuncture, premature ovarian insufficiency, diminished ovarian reserve, anxiety, depression, meta-analysis

## Abstract

**Background:**

The decreased ovarian function has a negative impact on the mental health of women and increases the risk of anxiety and depression. A growing number of clinical studies have demonstrated that acupuncture-related therapies can effectively and safely restore hormone levels and improve ovarian reserve function. However, the effectiveness of acupuncture-related therapies in alleviating anxiety and depression symptoms in patients with ovarian hypofunction has not been thoroughly evaluated. Therefore, this study conducted a systematic review and meta-analysis to assess the impact of the different acupuncture-related therapies on the mental health of patients with ovarian hypofunction.

**Methods:**

We comprehensively searched eight famous databases for randomized controlled trials up to October 30, 2024. Databases include PubMed, Web of Science, EMBASE and Cochrane Library, China Biomedical (CBM), China National Knowledge Infrastructure (CNKI), Wanfang Database and VIP Database.

**Results:**

The study included 12 RCTs, involving 780 patients with ovarian hypofunction, including 403 patients with POI, 297 patients with DOR, and 80 patients with POF. Acupuncture-related therapy was obviously superior to hormone therapy in relieving anxiety symptoms (SMD: -0.90; 95%CI: -1.28, -0.53; *P*<0.000 01) and depressive symptoms (SMD: -0.82; 95% CI: -1.25, -0.40; *P*=0.0001).

**Conclusions:**

Acupuncture-related therapy was more effective than hormone therapy in improving anxiety and depression symptoms in patients with ovarian hypofunction. This study supports the use of acupuncture-related therapies for women experiencing decreased ovarian function associated with mental health issues.

**Systematic review registration:**

https://www.crd.york.ac.uk/, **identifier CRD42023488015**.

## 1 Introduction

Ovarian function is a reflection of the quantity and quality of follicles in the ovary, serving as an indicator of women’s reproductive capacity. Ovarian aging is a persistent physiological process, with the gradual decrease in follicle numbers from birth to menopause (1). Menopause marks a natural stage in women’s lives, signifying follicular failure and the cessation of ovarian function (2). Epidemiological studies have reported that the average age for natural menopause in women is 51 years and 5 months (3). At present, the risk of premature ovarian function decline and early menopause in women around the world is gradually increasing. Premature ovarian insufficiency (POI) is defined as the onset of amenorrhea for more than 4 months before the age of 40 years, accompanied by elevated follicle-stimulating hormone (FSH) levels (4). Premature ovarian failure (POF) is considered as the terminal stage of POI (5). Although POF was reclassified as POI by the American Society for Reproductive Medicine (ASRM) in 2008, some studies still use the term POF (6). The prevalence rate of POI globally stands at 3.5%, particularly high in countries with low human development index (7, 8). Diminished ovarian reserve (DOR) is mainly manifested as the decline in the quality and quantity of oocytes and the decline in fertility but it is not postmenopausal, while there is no diagnostic criteria for DOR (9). Among various parameters used to diagnose DOR, FSH, antral follicle counting (AFC) and anti-Müllerian hormone (AMH) are widely recognized and commonly utilized standards at present (10). There is a clinical overlap between DOR and POI. Although the clinical manifestations of the two diseases are different, they have some common genetic mutations related to ovarian aging (1, 11, 12).

Decreased ovarian function occurs in both POI and DOR and causes short-term complications associated with menopausal symptoms, including hot flashes, night sweats, sleep disturbances, vaginal dryness, and fatigue, which have an impact on skeletal and cardiovascular health as well as decreased fertility and sexual function (13, 14). Evidence suggests that patients with POI and DOR are at higher risk for cardiovascular disease and osteoporosis compared to normal menopausal women (15–18). In addition to physiological effects, the loss of ovarian function also negatively impacts the mental health of patients, greatly reducing their productivity and quality of life, while increasing the risk of anxiety and depression (19, 20). Compared with the general population, women diagnosed with POI had higher levels of depression, perceived stress, and lower self-ratings (21). The anxiety and depression risks of POI patients in China are 4.62 times and 3.14 times higher than those of healthy women, while that of American patients is 6.67 times and 3.07 times higher than that of healthy women (22). Unfortunately, there is often neglect in clinical settings regarding the presence of these negative emotions in patients with ovarian hypofunction. Problems related to infertility and irregular menstruation are often prioritized in patients, while mental health is inadvertently ignored (23). Therefore, it is crucial to focus on providing comprehensive physiological and psychological treatment as well as preventive strategies for women with ovarian hypofunction (24).

Hormone replacement therapy (HRT) is the most commonly utilized treatment for POI and DOR, with potential benefits in alleviating sleep disorders but controversial effects on anxiety and depression (25, 26). Some studies suggest that HRT may not significantly improve negative emotions in patients (21, 27, 28). Furthermore, this treatment is associated with multiple side effects, an increased risk of heart disease, breast cancer, endometrial hyperplasia, and the formation of venous thromboembolism, and is not recommended for long-term use (29). In this context, acupuncture stand out from the numerous non-drug treatment (30). A substantial body of clinical studies has demonstrated the efficacy and safety of acupuncture in managing POI and DOR (31–33). Additionally, evidence-based research has also confirmed this view (34, 35). Acupuncture not only effectively enhances ovarian reserve function, promotes pregnancy, and restores hormone levels but also demonstrates positive effects in relieving anxiety and depression symptoms (36–39). However, most clinical studies have not observed the changes of patients’ mental health symptoms. Effectiveness of acupuncture in improving anxiety and depression symptoms of patients with ovarian dysfunction has not been evaluated. Therefore, in order to gain a better understanding of the impact of the different acupuncture-related therapies on the mental health and quality of life associated with ovarian hypofunction, we conducted a systematic review and meta-analysis of evidence from randomized controlled trials (RCTs) to assess the effectiveness of acupuncture-related therapies in alleviating anxiety and depression symptoms in patients with ovarian hypofunction.

## 2 Materials and methods

This study was conducted in accordance with the Preferred Reporting Items for Systematic Reviews and Meta-Analyses (PRISMA) (40) and PRISMA for acupuncture checklist (41). It was registered in PROSPERO, with the registration number CRD42023488015.

### 2.1 Search strategy

Two researchers (DN Z and XY L) comprehensively searched eight famous databases. It included four English databases: PubMed, Web of Science, EMBASE and Cochrane Library, and four Chinese databases: China Biomedical (CBM), China National Knowledge Infrastructure (CNKI), Wanfang Database and VIP Database. We retrieved articles before October 30, 2024, without language and regional restrictions. The complete search strategy for each database can be found in the **Supplementary Appendix**.

### 2.2 Inclusion criteria

The included studies met the following criteria:

1. The study type is randomized controlled trial (RCT), which is not limited by region or language.
2. Diagnosed as POI, POF or DOR. The diagnosis of POI meets the criteria for POI in the 2016 guidelines of the European Society of Human Reproduction and Embryology (4). Diagnostic criteria of DOR: ① FSH ≥ 10 mIU/mL after two examinations or FSH/LH > 2 mIU/mL; ②AMH < 1.1 ng/mL; ③ AFC < 7, and any two of the above three items can be diagnosed as DOR (42). The diagnostic criteria of POF refer to the POF guidelines stipulated by Gynecology Branch of Chinese Medical Association (43).
3. The subjects in the study scored the anxiety and/or depression scale before and after the intervention.
4. The intervention measures in the treatment group are acupuncture (ACU), electroacupuncture (EA), warming acupuncture (WA), moxibustion (MOX), acupoint catgut embedding (ACE) and the combination of various methods (such as ACU combined with EA, ACU combined with MOX, etc.). The intervention measures of the control group were estradiol hormone therapy.

### 2.3 Exclusion criteria

1. The course of treatment is less than one month.
2. No clear original data has been reported, or data cannot be extracted.
3. Duplicated published literature, or reported the same results.

### 2.4 Primary and secondary outcomes

We divided the result indicators into primary outcomes and secondary outcomes. At least one primary outcome indicator has been reported including: Self-rating Anxiety Scale (SAS) (44), Self-rating Depression Scale (SDS) (45), Hamilton Anxiety Scale (HAMA) (46) and Hamilton Depression Scale (HAMD) (47). Secondary outcomes were Kupperman index (KI), Integrals of traditional Chinese medicine (TCM) syndromes score and adverse events.

### 2.5 Study selection process and data extraction

Two researchers (SD H and DN Z) independently conducted literature screening and extract pertinent data. The screening process involved careful evaluation of titles, abstracts, and full texts to exclude articles that do not meet the predetermined inclusion and exclusion criteria. Consensus on the extracted data was reached through cross-checking. The following data were extracted from each study: first author’s name, publication year, age range of participants, sample size, diagnostic criteria, intervention measures for treatment and control groups, treatment duration, outcome indicators, and adverse events. Any discrepancies in this process were resolved through discussion and consultation with a third-party researcher (YZ).

### 2.6 Risk of bias assessment

Two researchers (SD H and DN Z) independently used the bias risk tool RoB2.0 recommended by Cochrane Manual to evaluate the bias risk of the included literature (48). The evaluation content includes the following six areas: bias caused by randomization process; Bias caused by deviation from intervention measures; Bias caused by lack of final data and bias of measurement results; Bias caused by choosing to report the results. Answer according to different questions set in each field (yes, probably yes, probably no, no, no information), and the evaluation results of each field are divided into low risk, some concerns and high risk. The differences in the evaluation process were solved by the third researcher (YZ).

### 2.7 Statistical analysis

We used RevMan 5.4 for meta-analysis. Each score is a numerical variable, and there may be different measurement methods and scoring standards. Therefore, we extracted the score difference before and after treatment, selected the standardized mean difference (SMD) as the effect index, and used the 95% confidence interval (95%CI) as the effect statistical test interval. Chi-square test and I-square test (I^2^) were used to evaluate the degree of heterogeneity. If I^2^>50%, *P*<0.10, the random-effect model is used for analysis; If I^2^ ≤ 50% and *P*≥0.10, the fixed-effect model is adopted. According to different disease diagnosis and different intervention measures, subgroup analysis was carried out to explore the potential sources of heterogeneity. We employed Stata 17.0 to conduct sensitivity analysis for exploring the robustness of the results. For a result indicator of more than 10 research reports, we conducted Egger test to evaluate the publication bias of the results.

## 3 Result

### 3.1 Study search and description

**Figure 1:**
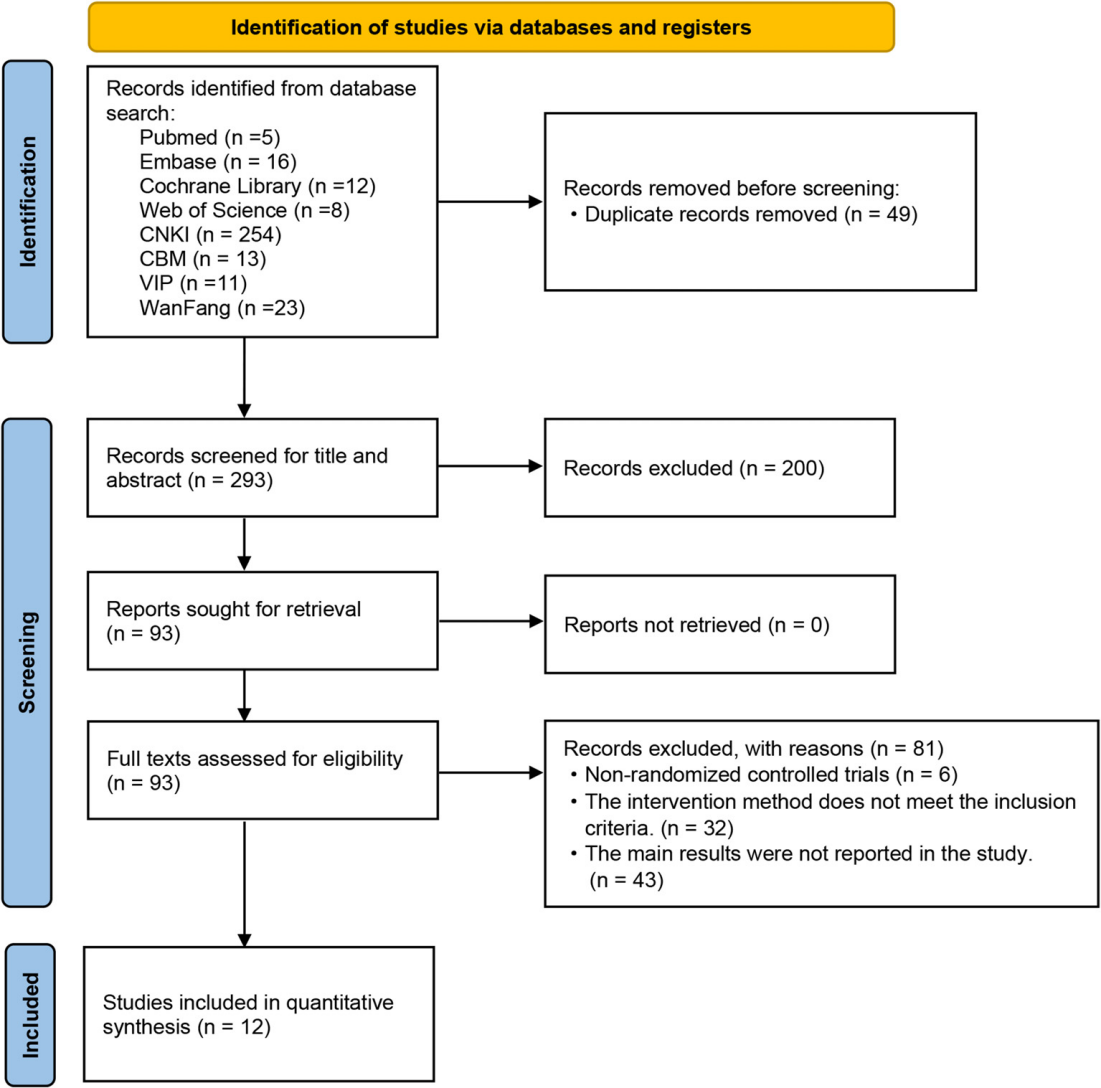
Flow diagram of studies included.

In the initial search, a total of 342 studies were retrieved. Subsequently, 49 duplicate studies were excluded, with 45 removed by software and 4 through manual review. After screening the titles and abstracts, an additional 200 studies were excluded. After thorough examination of the full texts by the researchers, a final count of 12 studies met our inclusion criteria, and the selection process is shown in **Figure 1**.

Among these 12 RCTs, one study was a three-arm trial, while the remaining studies were all two-arm trials. All the studies were published in Chinese and conducted within China. A total of 780 participants were enrolled, including 403 POI participants, 297 DOR participants and 80 POF participants. 375 participants were treated in the control group, taking estradiol; 405 cases received the intervention of the treatment group, including ACU, MOX, EA, ACE, as well as combinations of these methods. As for the primary outcome indicators, all 12 studies reported anxiety scores utilizing scales such as SAS scale in eleven trials and HAMA scale in one trial. Six studies reported the results of depression score, all of which used SDS scale. Eight studies documented adverse events occurring during their respective trials. The baseline characteristics and detailed acupuncture methodologies employed across these studies are presented in **Tables 1**, **2**.

**Table 1:** Baseline characteristics of the included studies.

References	Country	Diagnosis	Experimental			Control			Course	Type of outcomes
			Treatment	n	Age (Mean ± sd)	Treatment	n	Age (Mean ± sd)		
Bai et al. 2023 (49)	China	POI	ACU+MOX	30	31.40 ± 3.60	Climen	15	30.80 ± 5.50	12w	①②③④
Bai et al. 2023 (49)	China	POI	ACU	30	32.60 ± 5.80	Climen	15	30.80 ± 5.50	12w	①②③④
Dong 2021 (50)	China	DOR	ACE	19	34.00 ± 4.04	Femoston	18	34.17 ± 5.03	12w	③⑤
Du et al. 2022 (51)	China	POF	ACU+EA+MOX	40	32.50 ± 4.60	Progynova	40	32.00 ± 4.50	12w	①④
Liu 2021 (52)	China	POI	ACU+EA	21	34.36 ± 5.57	Femoston	22	34.44 ± 6.34	12w	①④
Luo et al. 2024 (95)	China	DOR	EA	30	34.67 ± 2.97	Femoston	30	34.47 ± 3.22	12w	①②⑤
Shi 2022 (53)	China	POI	ACE	30	31.60 ± 5.07	Femoston	30	32.16 ± 4.68	12w	①②⑤
Sun 2023 (54)	China	DOR	ACU+MOX	30	34.20 ± 2.64	Femoston	30	34.57 ± 3.29	12w	①⑤
Xu et al. 2021 (55)	China	POI	ACU	30	31.00 ± 4.00	Climen	30	29.00 ± 5.00	12w	①④
Xu 2023 (56)	China	DOR	ACU+EA+MOX	24	NR	Femoston	24	NR	8w	①②⑤
Xu et al. 2023 (57)	China	DOR	ACU+EA+MOX	16	31.63 ± 4.29	Femoston	16	30.88 ± 4.27	8w	①②
Yue et al. 2023 (58)	China	POI	ACU+EA	75	32.15 ± 5.02	Femoston	75	31.89 ± 4.87	12w	①②④⑤
Zhao 2024 (96)	China	DOR	MOX	30	36.93 ± 4.68	Femoston	30	36.23 ± 4.58	12w	①⑤

ACU, acupuncture; EA, electroacupuncture; MOX, moxibustion; ACE, acupoint catgut embedding; NR, No reported; W, week. ①SAS; ②SDS; ③HAMA; ④Kupperman Index; ⑤Integrals of TCM syndromes. Climen, compound packaging of estradiol valerate tablets(2mg)/estradiol cyproterone tablets(2mg:1mg); Femoston, compound packaging of estradiol valerate tablets(2mg)/estradiol cyproterone tablets(2mg:10mg); progynova, estradiol valerate tablets(1mg).

**Table 2:** Descriptions of the included acupuncture and related therapies.

References	Style of acupuncture	Names of acupuncture points used	Retention time		Acupuncture reaction	Frequency and course
Bai et al. 2023 (49)	ACU+MOX	Baihui(DU20), Shenting(DU24), Benshen(GB13), Tianshu(ST25), Zhongwan(CV12), Dahe(KI12), Zigong(EX-CA1), Guanyuan(CV4), Sanyinjiao(SP6), Taixi(KI3), Taichong(LR3), Shenshu(BL23), Shangliao(BL31), Ciliao(BL32), Zhongliao(BL33), Xialiao(BL34)	ACU+MOX	30min	Deqi	Five times a week for 12weeks
Bai et al. 2023 (49)	ACU	Baihui(DU20), Shenting(DU24), Benshen(GB13), Tianshu(ST25), Zhongwan(CV12), Dahe(KI12), Zigong(EX-CA1), Guanyuan(CV4), Sanyinjiao(SP6), Taixi(KI3), Taichong(LR3), Ciliao(BL32), Shenshu(BL23)	ACU	30min	Deqi	Five times a week for 12weeks
Dong 2021 (50)	ACE	Shenshu(BL23), Ganshu(BL18), Pishu(BL20), Huangshu(KI16), Luanchao, Zigong(EX-CA1), Sanyinjiao(SP6), Qihai(CV6), Xuehai(SP10), Ciliao(BL32), Guanyuan(CV4)	ACE	14days	Deqi	Once every two weeks for 12weeks
Du et al. 2022 (51)	ACU+EA+MOX	Baihui(DU20), Sishencong(EX-HN1), Shenting(DU24), Benshen(GB13), Shenmen(HT7), Neiguan(PC6), Zhongwan(RN12), Guanyuan(CV4), Qichong(ST30), Luanchao, Zusanli(ST36), Xuehai(SP10), Sanyinjiao(SP6), Taixi(KI3), Taichong(LR3), Ciliao(BL32), Shenshu(BL23)	ACU+EA MOX	30min 20min	Deqi	Three times a week for 12weeks
Liu 2021 (52)	ACU+EA	Guanyuan(CV4), Zigong(EX-CA1), Zusanli(ST36),Sanyinjiao(SP6), Taichong(LR3), Shenshu(BL23), Ciliao(BL32), Taixi(KI3)	ACU+EA	30min	Deqi	Three times a week for 12weeks
Luo et al. 2024 (95)	EA	Taichong(LR3), Ligou(LR5), Ququan(LR8), Jimai(LR12)	EA	30min	Deqi	Three times a week for 12weeks
Shi 2022 (53)	ACE	Zigong(EX-CA1), Guanyuan(CV4), Ganshu(BL18), Shenshu(BL23), Sanyinjiao(SP6)	ACE	15days	NR	Once every two weeks for 12weeks
Sun 2023 (54)	ACU+MOX	Baihui(DU20), Shenting(DU24), Yintang(EX-HN3), Sanyinjiao(SP6), Taichong(LR3), Taixi(KI3), Gongsun (SP4), Guanyuan(CV4), Dahe(KI12), Qixue(KI13)	ACU MOX	30min 1.5h	Deqi	Twice a week for 12 weeks
Xu et al. 2021 (55)	ACU	Baihui(DU20), Shenting(DU24), Benshen(GB13), Tianshu(ST25), Zhongwan(CV12), Dahe(KI12), Zigong(EX-CA1), Guanyuan(CV4), Sanyinjiao(SP6), Taixi(KI3), Taichong(LR3), Ciliao(BL32), Shenshu(BL23)	ACU	30min	Deqi	Five times a week for 12weeks
Xu 2023 (56)	ACU+EA+MOX	Guanyuan(CV4), Zhongwan(CV12), Tianshu(ST25), Ganshu(BL18), Pishu(BL20), Shenshu(BL23), Zusanli(ST36), Sanyinjiao(SP6), Taixi(KI3), Taichong(LR3), Qihai(CV6), Xuehai(SP10), Zigong(EX-CA1), Ciliao(BL32)	ACU+MOX EA	45min 15min	NR	Twice a week for 8 weeks
Xu et al. 2023 (57)	ACU+EA+MOX	Guanyuan(CV4), Zhongwan(CV12), Tianshu(ST25), Ganshu(BL18), Pishu(BL20), Shenshu(BL23), Zusanli(ST36), Sanyinjiao(SP6), Taixi(KI3), Taichong(LR3), Qihai(CV6), Xuehai(SP10), Zigong(EX-CA1), Ciliao(BL32)	ACU+MOX EA	45min 15min	NR	Twice a week for 8 weeks
Yue et al. 2024 (58)	ACU+EA	Tianshu(ST25), Quchi(LI11), Baihui(DU20), Taichong(LR3), Ganshu(BL18), Yanglingquan(GB34), Shenshu(BL23), Pishu(BL20), Guilai(ST29), Yintang(EX-HN 3), Xuehai(SP10), Ciliao(BL32), Diji(SP8), Sanyinjiao(SP6), Guanyuan(CV4), Zigong(EX-CA1), Dahe(KI12), Qihai(CV6), Benshen(BG13), Taixi(KI3), Huangshu(KI16), Zusanli(ST36), Zhongwan(CV12), Shenting(DU24), Yinlian(LR11)	ACU+EA	30min	Deqi	Three times a week for 12 weeks
Zhao 2024 (96)	MOX	Guanyuan(CV4), Sanyinjiao(SP6), Shenshu(BL23), Taixi(KI3)	MOX	NR	NR	Twice a week for 12 weeks

ACU, acupuncture; EA, electroacupuncture; MOX, moxibustion; ACE, acupoint catgut embedding; NR, No reported.

### 3.2 Quality assessment of included studies

**Figure 2:**
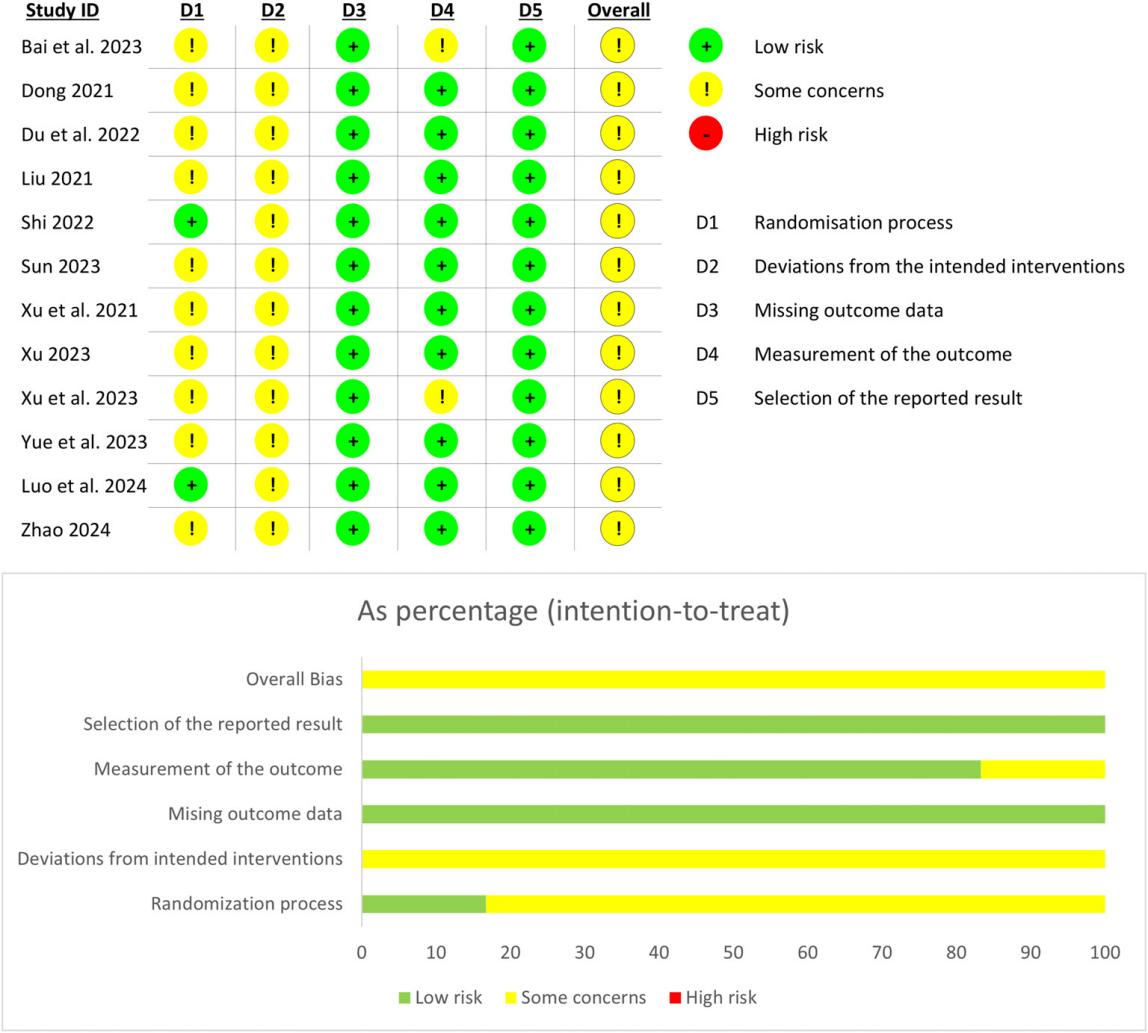
Risk of bias assessment.

All the studies mentioned the methods used in the process of random grouping, but ten studies did not describe whether the allocation sequence was hidden, so they were assessed as unclear. Because of the particularity of acupuncture operation (59), it is impossible to adopt blind method for both practitioners and subjects, so all the studies are assessed as some concerns. All included studies reported complete outcome data and were assessed as low risk. In two studies, it was found that there was a risk of bias in the measurement results, and the scoring standard and grade division were not clearly described. All the studies reported their predetermined results, and there was no selective reporting of results. The overall quality of the trial was assessed as moderate bias risk. The results of the risk evaluation are shown in **Figure 2**.

### 3.3 Evaluation of efficacy in anxiety

Eleven studies reported SAS score before and after treatment, while one study reported HAMA score. We extracted the difference in score values before and after treatment in these twelve studies for analysis, revealing a high level of heterogeneity (*P*<0.000 01, I^2^ = 83%). Consequently, we employed a random-effects model. The results indicated that acupuncture-related therapy was significantly more effective than hormone therapy in alleviating anxiety symptoms (SMD: -0.90; 95%CI: -1.28, -0.53; *P*<0.000 01).

**Figure 3:**
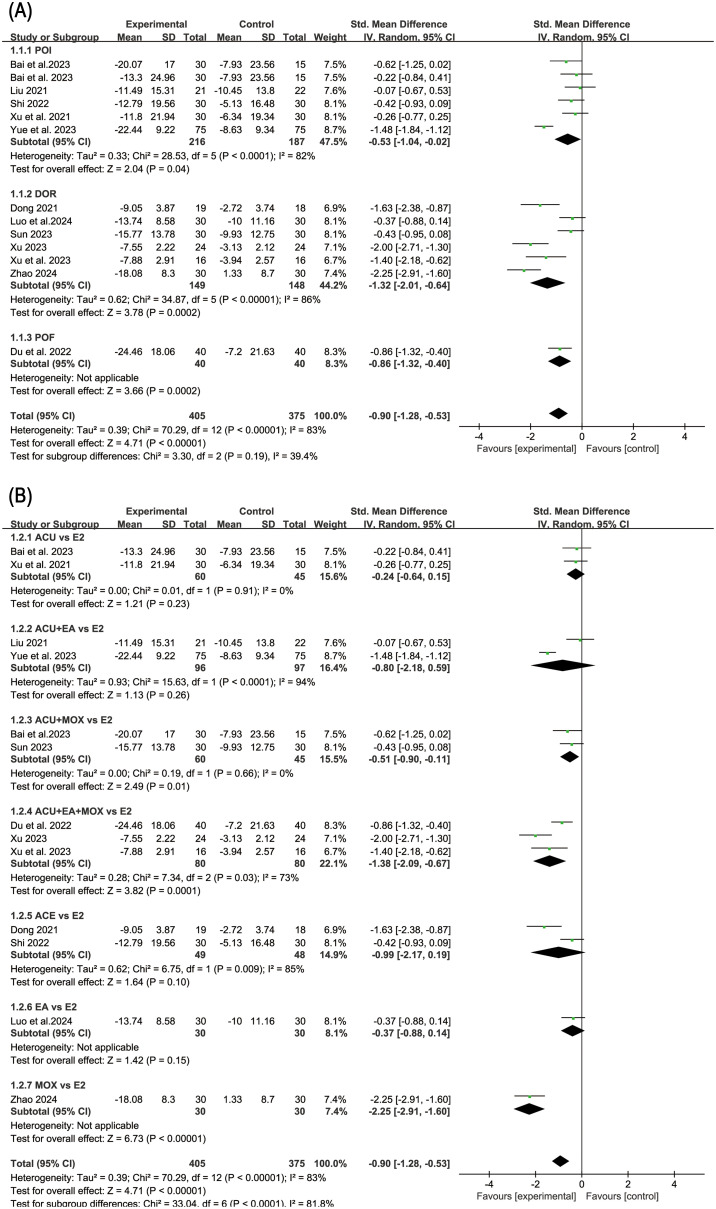
Forest plot of anxiety score in subgroup analyses. **(A)** Results of different disease subgroups; **(B)** Results of different intervention methods subgroups.

**Figure 4:**
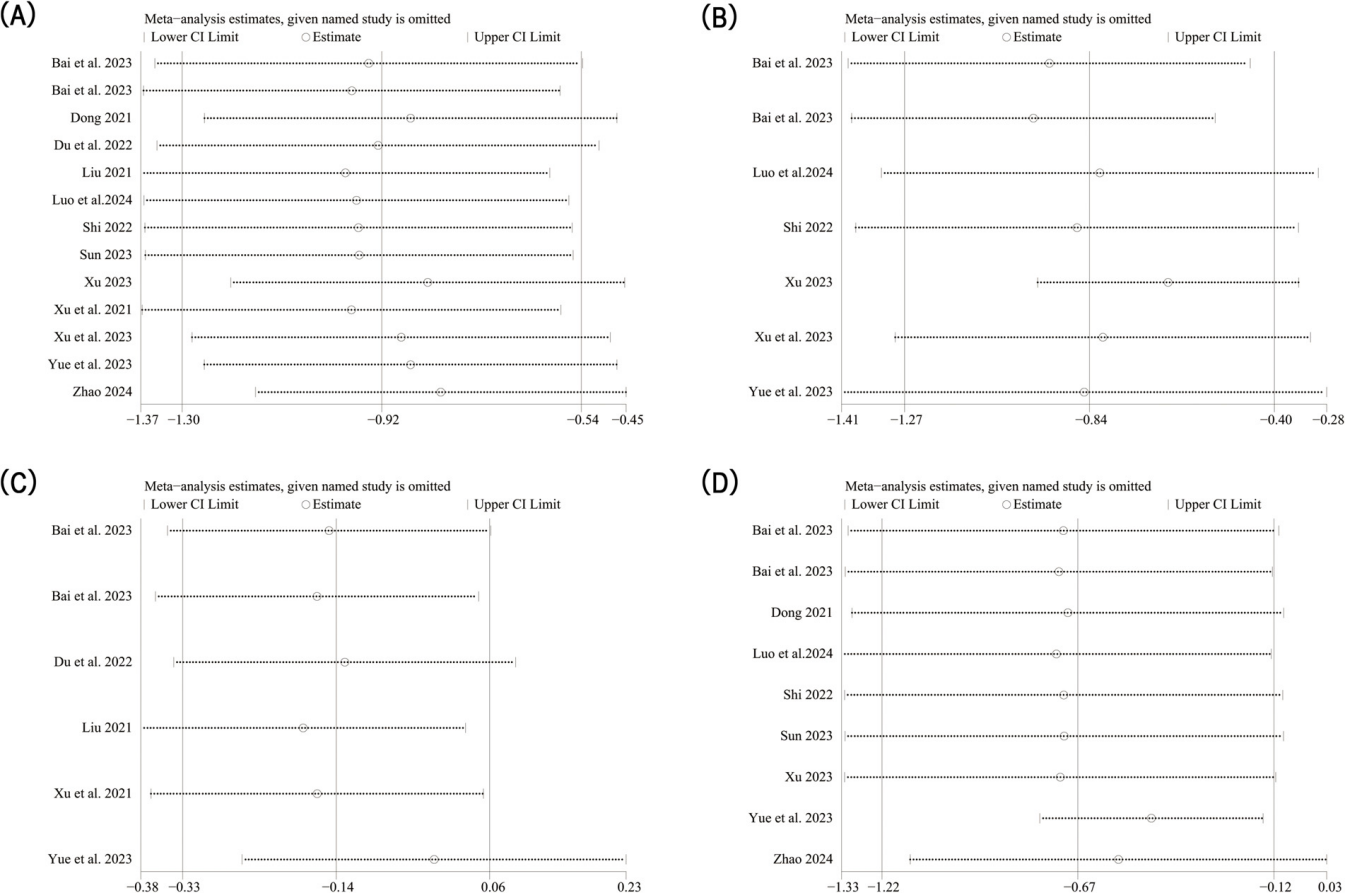
Sensitivity analysis results. **(A)** Sensitivity analysis of anxiety score; **(B)** Sensitivity analysis of depression score; **(C)** Sensitivity analysis of Kupperman index; **(D)** Sensitivity analysis of traditional Chinese medical syndrome integral.

In order to further explore the reasons for the high heterogeneity, we performed a subgroup analysis based on different diseases and various intervention measures. The results indicated that acupuncture-related therapies were more effective than hormone therapy in alleviating anxiety symptoms among patients with POI (SMD: -0.53; 95% CI: -1.04, -0.02; *P*=0.04), DOR (SMD: -1.32; 95% CI: -2.01, -0.64; *P*=0.0002), and POF (SMD: -0.86; 95% CI: -1.32, -0.40; *P*=0.0002) (**Figure 3A**). When comparing different interventions to hormone therapy, no statistically significant differences were found in improving anxiety symptoms among acupuncture (SMD: -0.24; 95% CI: -0.64, -0.15; *P*=0.23), acupuncture combined with electroacupuncture (SMD: -0.80; 95% CI: -2.18, -0.59; *P*=0.26), acupoint catgut embedding therapy (SMD: -0.99; 95%CI: -2.17, -0.19; *P*=0.10), and electroacupuncture (SMD: -0.37; 95% CI: -0.88, -0.14; *P*=0.15). However, in effecting relief of anxiety symptoms, acupuncture combined with moxibustion (SMD: -0.51; 95% CI: -0.90, -0.11; *P*=0.01) showed advantages over hormone therapy, as do acupuncture, electroacupuncture and moxibustion combined method (SMD: -1.38; 95% CI: -2.09, -0.67; *P*=0.0001) and moxibustion (SMD: -2.25; 95% CI: -2.91, -0.60; *P*<0.000 01) (**Figure 3B**). The results of sensitivity analysis were stable (**Figure 4A**).

### 3.4 Evaluation of efficacy in depression

**Figure 5:**
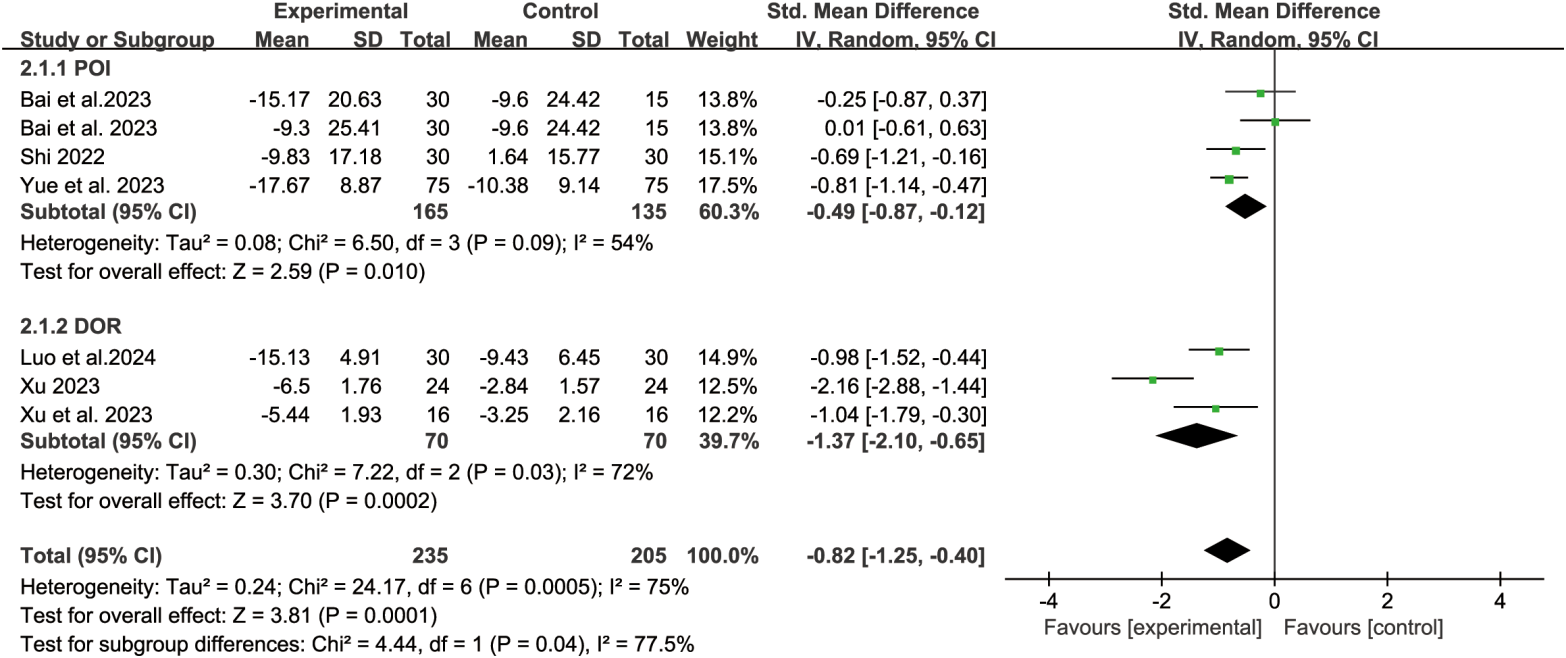
Forest plot of depression score in analysis of different disease subgroups.

Six studies reported SDS score. The random-effect model analysis (*P*=0.0001, I^2^ = 75%) showed that acupuncture-related therapy was significantly more effective than hormone therapy in alleviating depressive symptoms (SMD: -0.82; 95% CI: -1.25, -0.40; *P*=0.0001). Subgroup analysis of different diseases showed that acupuncture-related therapy was better than hormone therapy in improving depressive symptoms in both POI (SMD: -0.49; 95% CI: -0.87, -0.12; *P*=0.01) and DOR (SMD: -1.37; 95% CI: -2.10, -0.65; *P*=0.0002) (**Figure 5**). The results of sensitivity analysis were stable (**Figure 4B**).

### 3.5 KI and Integrals of TCM syndromes

**Figure 6:**
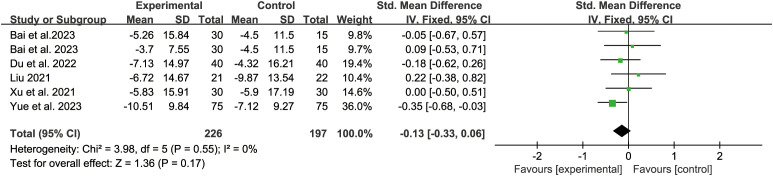
Forest plot of Kupperman Index.

Five studies reported the KI score. The fixed-effect model analysis (*P*=0.55, I^2^ = 0%) showed that there was no statistical difference between acupuncture-related therapy and hormone therapy (SMD: -0.13; 95% CI: -0.33, -0.06; *P*=0.17) (**Figure 6**). The results of sensitivity analysis were stable (**Figure 4C**).

**Figure 7:**
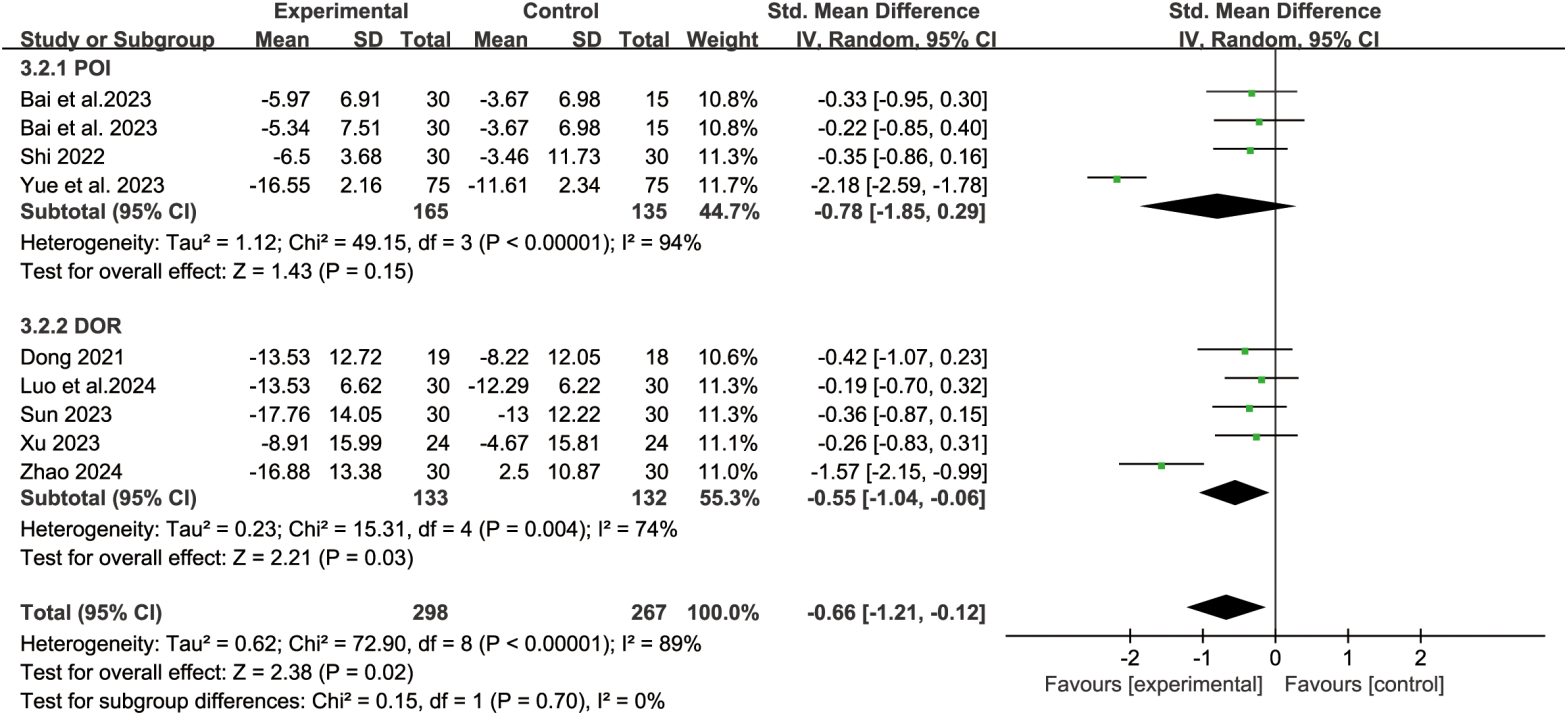
Forest plot of traditional Chinese medicine syndrome scores in analysis of different disease subgroups.

Eight studies reported the TCM syndrome scores. The random-effect model analysis (*P*<0.000 01, I^2^ = 89%) showed that acupuncture-related therapy was significantly more effective than hormone therapy in improving TCM syndromes (SMD: -0.66; 95% CI: -1.21, -0.12; *P*=0.02). Subgroup analyses of different diseases revealed that, in terms of enhancing TCM syndrome outcomes, acupuncture-related therapy outperformed hormone therapy for POI patients (SMD: -0.49; 95% CI: -0.87, -0.12; *P*=0.01). However, no significant difference was observed between the two therapies for DOR patients (SMD: -1.37; 95% CI: -2.10, -0.65; *P*=0.0002) (**Figure 7**). The results of sensitivity analysis were stable (**Figure 4D**).

### 3.6 Publication bias analysis

**Figure 8:**
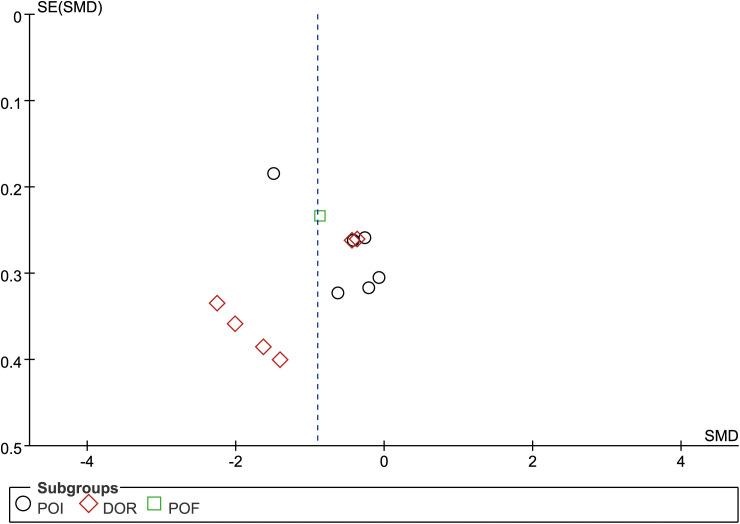
Funnel plot of anxiety score.

There are twelve studies that assessed participants’ anxiety scores, so evaluate this index with publication bias. First, we made a funnel chart with RevMan 5.4, but we couldn’t determine whether the funnel chart was symmetrical (**Figure 8**). Because the anxiety score is a continuous variable, we used the Egger test of Stata 17.0 to detect the publication bias (60). The results showed that *P*=0.734 > 0.05, which indicated that there was no publication bias.

### 3.7 Adverse

Eight studies reported adverse events. Among these, two studies indicated that patients did not experience any adverse events during the intervention. The adverse events observed in the remaining six studies are summarized in **Table 3**. According to the findings from these six studies, acupuncture-related therapies were associated with several adverse events, including needle sensation, subcutaneous congestion, allergic reactions, burns from moxibustion, and residual sensations. The adverse effects of hormone therapy primarily encompass nausea and vomiting, irregular vaginal bleeding, and slight palpitations.

**Table 3:** Adverse events.

References	Experimental		Control	
	Adverse events	n	Adverse events	n
Bai et al.2023 (49)	·Moxibustion allergy	2/30	·Irregular vaginal bleeding	2/30
	·Residual feeling after moxibustion	1/30		
	·Needle sensation remains after acupuncture	1/30		
Dong 2021 (50)	–	–	·Slight palpitation	2/18
Du et al.2022 (51)	·Subcutaneous congestion	2/40	·Anorexia	5/40
			·Vomiting	2/40
Liu 2021 (52)	·Subcutaneous congestion	1/22	·Nausea	1/22
			·Slight increases in liver function AL and AST were detected after 12 weeks of treatment	1/22
					Shi 2022 (53)	·Subcutaneous congestion	2/30	·Nausea	1/30
Zhao 2024 (96)	·Slight scald	2/30	–	–

n (number of events/total).

## 4 Discussion

This is the first systematic review and meta-analysis of acupuncture-related therapy in treating emotional disorders in patients with ovarian hypofunction. This meta-analysis included 780 participants with ovarian hypofunction from 12 RCTs. Our study compared the efficacy of acupuncture-related therapy and hormone therapy in alleviating symptoms of anxiety and depression symptoms in patients with ovarian hypofunction. The comprehensive findings indicate that acupuncture-related therapy outperforms hormone therapy in improving emotional disorders associated with this condition.

Anxiety and depression are prevalent emotional disorders that often co-occur (61). Women are at a higher risk of experiencing depression compared to men (62). The quality of life for infertile women is lower, accompanied by heightened levels of anxiety and depression (63). This situation is particularly pronounced among patients with ovarian hypofunction, who typically experience varying degrees of emotional disorders. Factors such as the patient’s desire for normal ovarian function and feelings of inadequacy stemming from unmet external expectations contribute significantly to their anxiety and depressive symptoms (64). Additionally, the decreased ability of the ovaries to work and hormone levels not only cause physical discomfort but also lead to anxiety and depression. These negative emotions interact with ovarian function in a mutually causal manner. Study have shown a positive correlation between the psychological state of POI patients and their ovarian function (65). Hence, it is imperative to prioritize the psychological well-being of these female patients. In today’s society, an increasing number of individuals seek professional treatment to alleviate symptoms related to anxiety and depression. To assist patients in accurately assessing the severity of their conditions, non-psychiatric physicians commonly employ tools such as the Depression Anxiety Rating Scale within clinical settings (66). In this study, Hamilton Anxiety Scale (HAMA), Hamilton Depression Scale (HAMD), Self-rated Anxiety Scale (SAS) and Self-rated Depression Scale (SDS) were selected as the primary outcome measures. These scales are currently recognized as valid instruments for assessing the severity of emotional disorders related to anxiety and depression (67, 68). Unfortunately, out of 12 RCTs included in this study, only one utilized the HAMA scale for assessing anxiety scores; there were no reports on results using the HAMD scale for measuring depression scores.

Acupuncture is a traditional Chinese medical method and a popular complementary alternative therapy. In China, it is commonly used to treat mental diseases such as anxiety, stress, depression, and insomnia (69). There is increasing evidence that acupuncture related-therapy is effective for anxiety and depression (70–73). Maunder found that more than 50 percent of patients with DOR needed to address mental health issues, and these patients said that acupuncture was a very beneficial form of treatment (74). According to traditional Chinese medicine, the pathogenesis of ovarian hypofunction combined with anxiety and depression mainly involves liver depression (energy imbalance). The therapies included in this study comprise acupuncture, electroacupuncture, moxibustion, and acupoint catgut embedding. These therapies stimulate specific acupoints and interact with the corresponding internal organs to facilitate the dredging of meridians, nourish the kidneys, strengthen the spleen, and harmonize liver function (thereby coordinating energy flow throughout the body). This approach aims to restore ovarian function and alleviate emotional distress (75–77). Western medicine believes that acupuncture-related therapy approaches may enhance the vitality of cranial nerves, alter the functional connectivity and structure of the brain, and thereby influence emotional regulation (78). The prefrontal cortex is a critical region of the brain involved in the regulation of anxiety and depressive emotions. Chen’s research has demonstrated that acupuncture therapy can enhance synaptic function and plasticity in neurons within the prefrontal cortex, promote neuronal signal transduction, modulate immune inflammatory responses, and consequently alleviate emotional disorders (79). Similarly, another research also indicates that the antidepressant effects of electroacupuncture are associated with an enhancement of synaptic transmission in the ventromedial prefrontal cortex (80). Previous research has shown that ovarian hormones have beneficial effects on neurobehavior, with increased estradiol levels alleviating anxiety in ovariectomized model (OVA) rats (81–83). Watcharin N’s research shows that progesterone inhibits depression and anxiety-like behavior by increasing lactobacillus flora in the intestinal flora of OVA mouse (84). Furthermore, the regulation of intestinal flora has been demonstrated by several studies to be a promising new intervention strategy for emotional disorders (85–87). It is worth noting that acupuncture-related therapy has a significant effect on improving the level of estradiol in patients with ovarian hypofunction (88–90). At the same time, these therapies have the potential to restore ovarian function by modulating intestinal flora (91, 92). Therefore, it is hypothesized that the mechanism of acupuncture improving emotional disorders in patients with ovarian hypofunction may be closely related to ovarian hormones and intestinal flora.

Our research results demonstrate that acupuncture-related therapy is beneficial for improving the emotional disorders of patients with ovarian hypofunction, with a low incidence of adverse effects. In combination with previous research evidence (34, 35, 93), acupuncture-related therapy shows advantages in restoring ovarian function without obvious adverse effects. We advocate for the consideration of acupuncture as an alternative treatment for ovarian hypofunction. However, there are some limitations to this study. Firstly, all included studies are from China, which may restrict the generalizability of our results due to the absence of patient data from other countries. Secondly, owing to the unique nature of acupuncture therapy, blinding methods cannot be implemented for subjects and intervention providers as they can be in pharmacological research. Additionally, most studies do not describe whether group concealment was carried out in the random assignment process, which leads to the risk of bias in these studies. Furthermore, our study selected acupuncture-related therapy as the intervention measure and included patients with various types of ovarian hypofunction diseases which may introduce heterogeneity into the analysis. To address these limitations and their potential impact on our findings, we conducted subgroup analyses based on different interventions and disease types within the experimental group. The results of subgroup analysis unfortunately do not indicate that the aforementioned factors are responsible for the high heterogeneity. Moreover, one study in the assessment of anxiety scores utilized the HAMA scale, which differed from the SAS scale used in other studies. While this discrepancy was initially suspected to be a contributing factor to the high heterogeneity, subsequent exclusion of the article did not result in any change in overall heterogeneity, leading us to dismiss this conjecture. However, the results of the scoring scale are derived from the subjective feelings of patients. Given the cognitive variations among individual patients and the potential adoption of different methodologies and scoring standards in result measurement, these factors may significantly influence the scoring outcomes. Therefore, we have extracted the difference in score values before and after treatment as our data and employ SMD for evaluating each result, facilitating synthesis and comparison of data. Lastly, due to a limited number of included studies, it is not feasible to further explore heterogeneity despite variations in acupuncture related-therapy such as acupoint selection, duration and course of treatment.

These limitations somewhat undermine the reliability of the evidence presented in the current study, so further verification is necessary to draw conclusive findings. We recommend designing more rigorous large-scale multi-center RCTs with pre-registration of clinical trial protocols to prevent duplication or selective reporting of anticipated research outcomes. Additionally, adherence to STRICTA guidelines (94) for reporting clinical trials on acupuncture treatment will contribute towards providing more dependable evidence-based medical information. Moreover, we encourage clinical therapists to prioritize not only addressing disease symptoms but also paying greater attention towards patients’ mental health.

## 5 Conclusion

The findings of this study indicate that acupuncture-related therapy has more advantages in improving anxiety and depression symptoms of patients with ovarian hypofunction than hormone therapy. However, our proposal is constrained by the limited number of randomized controlled trials included, which are also of low quality. We anticipate further multi-center and high-quality randomized controlled trials, to make up for the study to further prove the conclusion.

## Data Availability

The original contributions presented in the study are included in the article/**Supplementary Material**. Further inquiries can be directed to the corresponding authors.
