# Supplementary material for: Acupuncture and related therapies for anxiety and depression in patients with premature ovarian insufficiency and diminished ovarian reserve: a systematic review and meta-analysis
Source: Front Psychiatry. 2024 Dec 2;15:1495418. doi: 10.3389/fpsyt.2024.1495418 (PMC11647530; doi:10.3389/fpsyt.2024.1495418)
Supplement: Supplementary file 2 [file Table1.docx]

Appendix 1 search strategies

**Acupuncture and related therapies for anxiety and depression in patients with premature ovarian insufficiency and diminished ovarian reserve: a systematic review and meta-analysis**

Si-dan Huang^1&^, Dan-ni Zhang^1&^, Xu-liang Shi^12*^, Yi Zhang^1^, Xue-song Wang^1^, Yan-fen She^12^,Ce Liang^3^, Xin-yue Li^1^

1 School of Acupuncture-Moxibustion and Tuina, Hebei University of Chinese Medicine, Shijiazhuang, Hebei, China

2 Hebei International Joint Research Center for Dominant Diseases in Chinese Medicine and Acupuncture, Hebei University of Chinese Medicine, Shijiazhuang, Hebei, China

3 Pharmacological Lab of Traditional Chinese Medicine, Hebei University of Chinese Medicine, Hebei TCM Formula Granule Technology Innovation Center, Shijiazhuang, Hebei, China

& These authors contributed equally to this work.

* Corresponding authors: E-mail addresses: shi-xu-liang@163.com (XL S).

**Appendix 1. The search strategies for the included RCTs for Pubmed.**

#1 (((((Acupuncture[MeSH Terms]) OR (Acupuncture, Ear[MeSH Terms])) OR (Acupuncture Therapy[MeSH Terms])) OR (Acupuncture Points[MeSH Terms])) OR (Moxibustion[MeSH Terms])) OR (Auriculotherapy[MeSH Terms])

#2 ((((((((((((((((((((((((Auriculotherapy[Title/Abstract]) OR (needle-warming moxibustion[Title/Abstract])) OR (acupoint[Title/Abstract])) OR (acupoint stimulation[Title/Abstract])) OR (acupoint therapy[Title/Abstract])) OR (needling[Title/Abstract])) OR (needle therapy[Title/Abstract])) OR (fire needle[Title/Abstract])) OR (fire needling[Title/Abstract])) OR (electroacupuncture[Title/Abstract])) OR (electro-acupuncture[Title/Abstract])) OR (acupuncture treatment[Title/Abstract])) OR (acupuncture treatments[Title/Abstract])) OR (moxibustion[Title/Abstract])) OR (de qi[Title/Abstract])) OR (Meridian[Title/Abstract])) OR (plum blossom needle[Title/Abstract])) OR (blood letting therapy[Title/Abstract])) OR (scalp acupuncture[Title/Abstract])) OR (auricular acupuncture[Title/Abstract])) OR (auriculotherapy[Title/Abstract])) OR (needle-free injection[Title/Abstract])) OR (acupoint embedding therapy[Title/Abstract])) OR (catgut implantation at acupoint[Title/Abstract])) OR (acupoint application[Title/Abstract])

#3 #1 OR #2

#4 (Primary Ovarian Insufficiency[MeSH Terms]) OR (Ovarian Reserve[MeSH Terms])

#5 (((((((premature ovarian failure[Title/Abstract]) OR (primary ovarian insufficiency[Title/Abstract])) OR (premature ovarian insufficiency[Title/Abstract])) OR (decreased ovarian reserve[Title/Abstract])) OR (diminished ovarian reserve[Title/Abstract])) OR (POI[Title/Abstract])) OR (POF[Title/Abstract])) OR (DOR[Title/Abstract])

#6 #4 OR #5

#7 ((anxiety[MeSH Terms]) OR (depression[MeSH Terms])) OR (depressive disorder[MeSH Terms])

#8 (((anxiety[Title/Abstract]) OR (depression[Title/Abstract])) OR (depressive symptom[Title/Abstract])) OR (depressive symptom[Title/Abstract])

#9 #7 OR #8

#10 (Randomized Controlled Trials[MeSH Terms]) OR (Randomized Controlled Trials as Topic[MeSH Terms])

#11 ((Randomized Controlled Trial[Title/Abstract]) OR (RCT[Title/Abstract])) OR (random[Title/Abstract])

#12 #10 OR #11

#13 #3 AND #6 AND #9 AND #12

**Appendix 1. The search strategies for the included RCTs for EMBASE.**

#1 'acupuncture'/exp OR 'acupuncture, ear'/exp OR 'acupuncture therapy'/exp OR 'acupuncture points'/exp OR 'moxibustion'/exp OR 'auriculotherapy'/exp OR 'warm needle acupuncture':ti,ab,kw OR 'needle-warming moxibustion':ti,ab,kw OR acupoint:ti,ab,kw OR 'acupoint stimulation':ti,ab,kw OR 'acupoint therapy':ti,ab,kw OR needling:ti,ab,kw OR 'needle therapy':ti,ab,kw OR 'fire needle':ti,ab,kw OR 'fire needling':ti,ab,kw OR electroacupuncture:ti,ab,kw OR 'electro acupuncture':ti,ab,kw OR 'acupuncture treatment':ti,ab,kw OR 'acupuncture treatments':ti,ab,kw OR moxibustion:ti,ab,kw OR 'de qi':ti,ab,kw OR meridian:ti,ab,kw OR 'plum blossom needle':ti,ab,kw OR 'blood letting therapy':ti,ab,kw OR 'scalp acupuncture':ti,ab,kw OR 'auricular acupuncture':ti,ab,kw OR auriculotherapy:ti,ab,kw OR 'needle-free injection':ti,ab,kw OR 'acupoint embedding therapy':ti,ab,kw OR 'catgut implantation at acupoint':ti,ab,kw OR 'acupoint application':ti,ab,kw

#2 'primary ovarian insufficiency'/exp OR 'ovarian reserve'/exp OR 'premature ovarian failure':ti,ab,kw OR 'primary ovarian insufficiency':ti,ab,kw OR 'premature ovarian insufficiency':ti,ab,kw OR 'decreased ovarian reserve':ti,ab,kw OR 'diminished ovarian reserve':ti,ab,kw OR poi:ti,ab,kw OR pof:ti,ab,kw OR spine:ti,ab,kw

#3 'anxiety'/exp OR 'depression'/exp OR 'depressive disorder'/exp OR anxiety:ti,ab,kw OR depression:ti,ab,kw OR 'depressive symptom':ti,ab,kw OR 'depressive disorder':ti,ab,kw

#4 'randomized controlled trials'/exp OR 'randomized controlled trials as topic'/exp OR 'randomized controlled trial':ti,ab,kw OR random:ti,ab,kw OR rct:ti,ab,kw

#5 #1 AND #2 AND #3 AND #4

**Appendix 1. The search strategies for the included RCTs for Cochrane Library.**

#1 MeSH descriptor: [Acupuncture] explode all trees

#2 MeSH descriptor: [Acupuncture, Ear] explode all trees

#3 MeSH descriptor: [Acupuncture Therapy] explode all trees

#4 MeSH descriptor: [Acupuncture Points] explode all trees

#5 MeSH descriptor: [Moxibustion] explode all trees

#6 MeSH descriptor: [Auriculotherapy] explode all trees

#7 (warm needle acupuncture or needle-warming moxibustion or acupoint or acupoint stimulation or acupoint therapy or needling or needle therapy or fire needle or fire needling or electroacupuncture or electro-acupuncture or acupuncture treatment or acupuncture treatments or moxibustion or de qi or Meridian or plum blossom needle or blood letting therapy or scalp acupuncture or auricular acupuncture or auriculotherapy or needle-free injection or acupoint embedding therapy or catgut implantation at acupoint or acupoint application):ti,ab,kw

#8 #1 or #2 or #3 or #4 or #5 or #6 or #7

#9 MeSH descriptor: [Primary Ovarian Insufficiency] explode all trees

#10 MeSH descriptor: [Ovarian Reserve] explode all trees

#11 (premature ovarian failure or primary ovarian insufficiency or premature ovarian insufficiency or decreased ovarian reserve or diminished ovarian reserve or POI or POF or DOR):ti,ab,kw

#12 #9 or #10 or #11

#13 MeSH descriptor: [Anxiety] explode all trees

#14 MeSH descriptor: [Depression] explode all trees

#15 MeSH descriptor: [Depressive Disorder] explode all trees

#16 (anxiety or depression or depressive symptom or depressive disorder):ti,ab,kw

#17 #13 or #14 or #15 or #16

#18 MeSH descriptor: [Randomized Controlled Trial] explode all trees

#19 MeSH descriptor: [Randomized Controlled Trials as Topic] explode all trees

#20 (Randomized Controlled Trial or random or RCT):ti,ab,kw

#21 #18 or #19 or #20

#22 #8 and #12 and #17 and #21

**Appendix 1. The search strategies for the included RCTs for** **Web of Science.**

#1 ((((((((((((((((((((((((((((((TS=(Acupuncture)) OR TS=(Acupuncture, Ear)) OR TS=(Acupuncture Therapy)) OR TS=(Acupuncture Points)) OR TS=(Moxibustion)) OR TS=(Auriculotherapy)) OR TI=(warm needle acupuncture)) OR TI=(needle-warming moxibustion)) NOT TI=(acupoint)) OR TI=(acupoint stimulation)) OR TI=(acupoint therapy)) OR TI=(needling)) OR TI=(needle therapy)) OR TI=(fire needle)) OR TI=(fire needling)) OR TI=(electroacupuncture)) OR TI=(electro-acupuncture)) OR TI=(acupuncture treatment)) OR TI=(acupuncture treatments)) OR TI=(moxibustion)) OR TI=(de qi)) OR TI=(Meridian)) OR TI=(plum blossom needle)) OR TI=(blood letting therapy )) OR TI=(scalp acupuncture)) OR TI=(auricular acupuncture)) OR TI=(auriculotherapy)) OR TI=(needle-free injection)) OR TI=(acupoint embedding therapy)) OR TI=(catgut implantation at acupoint)) OR TI=(acupoint application)

#2 (((((((((TS=(Primary Ovarian Insufficiency)) OR TS=(Ovarian Reserve)) OR TI=(premature ovarian failure)) OR TI=(primary ovarian insufficiency)) OR TI=(premature ovarian insufficiency)) OR TI=(decreased ovarian reserve)) OR TI=(diminished ovarian reserve)) OR TI=(POI)) OR TI=(POF)) OR TI=(DOR)

#3 ((((((TS=(anxiety)) OR TS=(depression)) OR TS=(depressive disorder)) OR AB=(anxiety)) OR AB=(depression)) OR AB=(depressive symptom)) OR AB=(depressive disorder)

#4 ((((TS=(Randomized Controlled Trials as Topic)) OR TI=(Randomized Controlled Trial)) OR AB=(Randomized Controlled Trial)) OR AB=(RCT)) OR AB=(random)

#5 #1 AND #2 AND #3 AND #4

**Appendix 1. The search strategies for the included RCTs for** **China National Knowledge Infrastructure (CNKI)**

SU='针灸'+'针刺'+'电针'+'头针'+'体针'+'腹针'+'梅花针'+'火针'+'温针灸'+'艾灸'+'灸法'+'耳穴'+'耳针'+'针药联合'+'穴位注射'+'穴位埋线'+'穴位敷贴' AND SU='早发性卵巢功能不全'+'卵巢早衰'+'POI'+'POF'+'卵巢储备功能减退'+'卵巢储备功能下降'+'DOR' AND FT='焦虑'+'抑郁'+'SAS'+'SDS'+'HAMA'+'HAMD' AND FT='随机'

**Appendix 1. The search strategies for the included RCTs for Wanfang Data**

题名或关键词:(针灸 or 针刺 or 电针 or 温针 or 头针 or 体针 or 腹针 or 梅花针 or 火针 or 温针灸 or 艾灸 or 灸法 or 耳穴 or 耳针 or 针药联合 or 穴位注射 or 穴位埋线 or 穴位敷贴) and 题名或关键词:(早发性卵巢功能不全 or 卵巢早衰 or POI or POF or 卵巢储备功能减退 or 卵巢储备功能下降 or DOR) and 全部:(焦虑 or 抑郁 or SAS or SDS or HAMA or HAMD) and 全部:(随机)

**Appendix 1. The search strategies for the included RCTs for** **Chinese Scientific Journal Database (VIP)**

M=(针灸 OR 温针灸 OR 电针 OR 针刺 OR 针法 OR 艾灸 OR 灸法 OR 耳穴 OR 耳压 OR 耳埋 OR 耳针 OR 梅花针 OR 七星针 OR 火针 OR 燔针 OR 穴位贴敷 OR 穴位敷贴 OR 穴位注射 OR 穴位埋线) AND M=(卵巢早衰 OR 卵巢功能早衰 OR 早绝经 OR 早发性卵巢功能不全 OR 卵巢功能减退 OR 卵巢衰竭 OR 卵巢低储备 OR 卵巢储备功能减退 OR 卵巢储备功能不良 OR 卵巢储备功能下降 OR 卵巢功能低下 OR 卵巢储备功能降低 OR 卵巢储备功能低下) AND U=(焦虑 OR 抑郁 OR HAMA OR HAMD) AND U=(随机)**Appendix 1. The search strategies for the included RCTs for China Biology Medicine (CBM)**

("针灸"[常用字段:智能] OR "针刺"[常用字段:智能] OR "电针"[常用字段:智能] OR "头针"[常用字段:智能] OR "体针"[常用字段:智能] OR "腹针"[常用字段:智能] OR "梅花针"[常用字段:智能] OR "火针"[常用字段:智能] OR "耳针"[常用字段:智能] OR "艾灸"[常用字段:智能] OR "灸法"[常用字段:智能] OR "温针灸"[常用字段:智能] OR "耳穴"[常用字段:智能] OR "针药并用"[常用字段:智能] OR "穴位注射"[常用字段:智能] OR "穴位埋线"[常用字段:智能] OR "穴位贴敷"[常用字段:智能]) AND ("原发性卵巢功能不全"[不加权:扩展] OR "卵巢储备功能"[不加权:扩展]) AND ("焦虑"[全部字段:智能] OR "抑郁"[全部字段:智能] OR "表现焦虑量表"[不加权:扩展] OR "患者健康问卷"[不加权:扩展]) AND ("随机"[全部字段:智能] OR "随机对照试验"[不加权:扩展])
